# Immune Regulatory and Underlying Mechanisms of Polysaccharides from *Aronia melanocarpa* Fruit by Integrated Analysis of Multiple Endogenous Metabolism

**DOI:** 10.3390/molecules31071166

**Published:** 2026-04-01

**Authors:** Jinxu Dong, Honglei Liu, Lei Wang, Yan Liu, Xin Huang

**Affiliations:** 1 Chromatography and Mass Spectrometry Technology Platform, Jilin Ginseng Academy, Changchun University of Chinese Medicine, Changchun 130117, China; 2 Jinuo Biological Engineering Co., Ltd., Changchun 130600, China; 3 Institute of Special Animal and Plant Sciences, Chinese Academy of Agricultural Sciences, Changchun 130112, China

**Keywords:** *Aronia melanocarpa* fruit polysaccharide, immune regulation mechanism, integrated multiple metabolomics and lipidomics, humoral and cellular immunity responses, oxidative damage

## Abstract

The fruit of *Aronia melanocarpa* (Michx.) Elliott is a berry with multiple properties and was included as a new raw food material by the National Health Commission of China (NHC) in September 2018. This study focused on the immune regulatory properties and underlying mechanism of polysaccharides extracted from *Aronia melanocarpa* fruit (AMFP) by undertaking an integrated analysis of multiple endogenous metabolic pathways. An improvement in AMFP in immunosuppressed model mice at three levels of immune organs, immune cells, and immune factors was determined. The immunomodulatory role of AMFP was assessed through measurement of metabolomic and lipidomic profilings by UPLC-Q-TOF/MS. A total of 53 differential endogenous metabolites in the urinary, serum, and lipid metabolomics were identified, followed by KEGG pathway enrichment. Furthermore, the underlying mechanisms were elucidated by an integrated analysis of multiple metabolomics and lipidomics. Primarily, we found regulation of immune-related metabolic pathways, including nicotinate and nicotinamide metabolism, sphingolipid metabolism, glycerophospholipid metabolism, purine metabolism, steroid hormone biosynthesis, and arachidonic acid metabolism. The results also demonstrated the mutual validation of key pathways and mechanisms. AMFP potentiated both humoral and cellular immunity responses and protected the immune system from oxidative damage. This research provides a reference and a basis for the development and application of AMFP in the field of health foods that regulate immunity.

## 1. Introduction

The fruit of *Aronia melanocarpa* (Michx.) Elliott is a spherical berry with deep purple to dark red skin, flesh, and seeds [1]. *Aronia melanocarpa* fruit was included as a new food raw material by the National Health Commission of China (NHC) in September 2018 [2]. The main constituents of the *Aronia melanocarpa* fruit are polyphenols, organic acids, flavonoids, polysaccharides, and other bioactive compounds [3,4]. *Aronia melanocarpa* fruit exhibits multiple properties [5,6,7,8,9,10,11,12], with the antioxidant activity in particular being 2.5-fold higher than that of blueberries and 9-fold higher compared with cranberries [13]. Current studies have revealed that polysaccharides from *Aronia melanocarpa* fruit exerted antioxidant [14,15], anti-inflammatory [16], anti-aging [16], immunomodulatory [17], and liver fibrosis-alleviating [18] effects through modulation of the gut microbiota. It is well known that natural polysaccharides exhibit a broad spectrum of bioactivities, with immunomodulation being the most prominent.

Metabolomics was first proposed by Nicholson’s team in 1999 [19]. It is a scientific discipline that investigates biological systems by examining changes in metabolic products following stimulation or perturbation. Metabolomics, as a branch of systems biology, is characterized by several advantages: metabolic products can directly reflect changes occurring within an organism; alterations in genes, proteins, and other biomolecules can be amplified and thus more readily observed through changes in metabolic products; the data are relatively easy to analyze; and sampling is convenient. Compared with other omics technologies, metabolomics can better reflect the holistic information of an organism. Metabolites represent the terminal products of biochemical regulation within biological systems, reflecting biological events that have already occurred. The effects of gene expression and protein alterations on biological systems are ultimately reflected at the metabolomic level. The most widely used metabolomics methods at present are nuclear magnetic resonance spectroscopy (NMR) and mass spectrometry (MS) combined with gas chromatography (GC), liquid chromatography (LC), or capillary electrophoresis (CE). Comprehensive qualitative and quantitative analysis of endogenous differential metabolites in organisms can elucidate the pathophysiological changes in complex diseases and pharmacological intervention mechanisms at the molecular level [20,21].

Therefore, the aim of this study is to characterize the immune regulatory and underlying mechanisms of polysaccharides from *Aronia melanocarpa* fruit (AMFP). Using ultra-performance liquid chromatography coupled with a quadrupole time-of-flight mass spectrometry (UPLC-Q-TOF/MS)-based omics approach, metabolomic and lipidomic analyses were performed to assess the impact of AMFP on metabolites in immunosuppressed mice. Differential metabolite analysis and pathway enrichment were systematically explored. Furthermore, by undertaking an integrated metabolomics technology and multi-dimensional omics data approach, a more comprehensive and precise elucidation of the immunomodulatory mechanisms of AMFP was achieved. This research provides a reference and a basis for the development and application of AMFP in the field of health foods that regulate immunity.

## 2. Results and Discussion

### 2.1. Immunomodulatory Effects of AMFP in Immunosuppressed Mice

#### 2.1.1. Effect of AMFP on Immune Organ Index

Body weight serves as one of the intuitive indicators of overall health status in mice. The spleen and thymus are critical immune organs, as their weights reflect the quantity of immune cells and the immune capacity. As shown in Table S1, compared with the C group, the M group exhibited significantly decreased body weight and immune organ index (*p* < 0.05), indicating the successful establishment of the immunosuppressed mouse model. Compared with the M group, the A group showed significant increases in body weight, spleen index, and thymus index (*p* < 0.05), with immune organ status approaching that of the C group. This indicated that AMFP exhibited an alleviating effect on the cyclophosphamide-induced atrophy of the spleen and thymus, thus promoting the development of immune organs.

#### 2.1.2. Effect of AMFP on Delayed-Type Hypersensitivity

As shown in Table S1, the auricle swelling degree in the M group was significantly higher than that in the C group (*p* < 0.05). Compared with the M group, the A group showed significant inhibition of the auricle swelling degree (*p* < 0.01), suggesting AMFP could alleviate delayed-type hypersensitivity in immunosuppressed mice and enhance the body’s immune response.

#### 2.1.3. Effect of AMFP on Peripheral Blood Parameters

Compared with the C group, the M group showed significantly decreased counts of white blood cells, platelets, and red blood cells, as well as reduced percentages of neutrophils, lymphocytes, and monocytes (*p* < 0.05), as shown in Table S1. And compared with the M group, all the peripheral blood parameters showed a significant increase in the A group (*p* < 0.05) and tended toward a normal level. AMFP could enhance the cellular immune function of immunocompromised mice by upregulating peripheral blood cells.

#### 2.1.4. Effect of AMFP on Phagocytosis of Macrophages

Macrophage phagocytosis could reflect the body’s non-specific immune function. In the carbon clearance assay, carbon particles in the blood could be phagocytosed and cleared by macrophages. Therefore, the clearance rate of carbon particles in blood can assess the phagocytic ability of macrophages. As shown in Table S1, the phagocytic index was significantly lower in the M group (*p* < 0.05). This suggested that cyclophosphamide reduced the phagocytosis of macrophages. Compared with the M group, the carbon clearance index and phagocytic index of the A group were significantly increased (*p* < 0.05), suggesting that AMFP enhanced macrophage phagocytosis in immunocompromised mice.

#### 2.1.5. Effect of AMFP on Immune Cytokines IL-2 and IFN-γ

IL-2 mediates humoral immune responses. IFN-γ has functions such as activation, induction, and promotion of the immune cell differentiation. As shown in Table S1, the serum levels of IL-2 and IFN-γ were significantly lower in the M group than in the C group (*p* < 0.01). The serum IL-2 level was significantly elevated in the A group (*p* < 0.05), with an even more significant increase in the IFN-γ level (*p* < 0.01). AMFP restored immune function and alleviated cyclophosphamide-induced immunosuppression by upregulating serum IL-2 and IFN-γ levels.

#### 2.1.6. Effect of AMFP on Antioxidant Activity

Reactive oxygen species (ROS) are involved in immune responses, and their imbalance induces oxidative damage, leading to immune deficiency. MDA, SOD and GSH-Px are widely used biomarkers for assessing antioxidant activity in vivo. As shown in Table S1, compared with the C group, the M group showed a significantly higher MDA level (*p* < 0.05), whereas the SOD and GSH-Px activities were significantly lower (*p* < 0.01). The MDA level in the A group was significantly decreased (*p* < 0.01), and the SOD and GSH-Px activities were significantly increased (*p* < 0.01). The results demonstrated that AMFP significantly enhanced serum antioxidant activities, thereby improving ROS scavenging capacity.

#### 2.1.7. Effect of AMFP on Splenic Lymphocyte Proliferation

Splenic lymphocyte proliferation is a commonly used indicator to reflect cellular immune function. The proliferative capacity of splenic lymphocytes was significantly decreased in the M group vs. the C group (*p* < 0.01) (Table S1), and in the A group, it was significantly increased vs. the M group (*p* < 0.05). AMFP could improve the immune function of immunocompromised mice by enhancing splenic lymphocyte proliferation.

Based on the in vivo experiments using model mice, at the three levels of immune organs, immune cells, and immune factors, the results demonstrated that AMFP acted on the immune system. By promoting the proliferation of immune organs, enhancing the proliferation of immune cells, and stimulating the secretion of immune cytokines, AMFP exerted both non-specific and specific immunomodulatory effects. At the same time, AMFP exerted antioxidant activity in vivo to reduce the damage to the immune system caused by peroxidative damage, thus indirectly regulating immune function. A potential regulatory relationship existed between antioxidant activity and immunomodulatory effects, and this mechanism could be elucidated through subsequent molecular-level studies.

### 2.2. Urinary Metabolomics Revealed the Immunomodulatory Effects of AMFP

#### 2.2.1. Metabolomics Data Collection from Urine Samples

UPLC-Q-TOF/MS analysis of urine samples revealed different metabolites in the Base Peak Ion (BPI) chromatogram among the C, M, and A groups in both positive and negative ion modes (Figure S1). The collected metabolomics data were processed and imported for multivariate statistical analysis.

#### 2.2.2. Multivariate Statistical Analysis of Urinary Metabolomics

**Figure 1:**
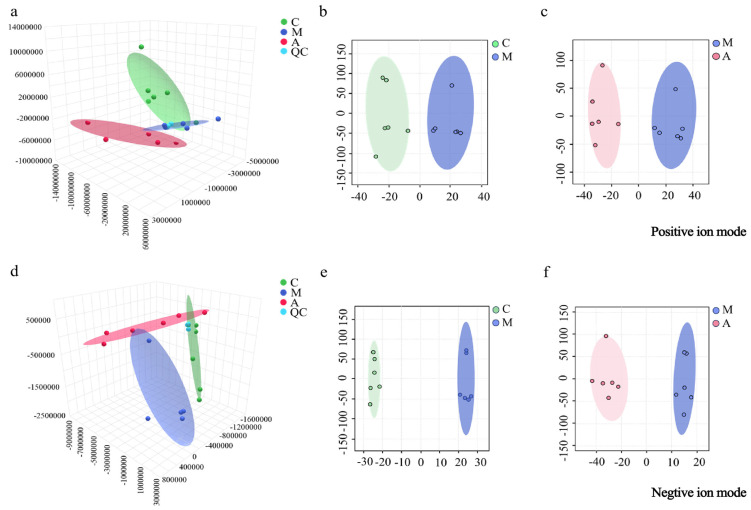
PCA 3D plots of urine samples from the C, M, and A groups in positive (**a**) and negative (**d**) ion modes; OPLS-DA plots of urine samples from the C and M groups in positive (**b**) and negative (**e**) ion modes; OPLS-DA plots of urine samples from the M and A groups in positive (**c**) and negative (**f**) ion modes.

The urinary metabolomics data were screened and analyzed by PCA and OPLS-DA. As shown in Figure 1a,d, PCA revealed that within each group, the metabolic profiles were relatively clustered, and significant separation was observed between the groups in positive and negative ion modes. This indicated that there were notable differences in the physiological and metabolic states among the groups. OPLS-DA eliminated irrelevant variables and identified discriminatory variables (VIP > 1.0) of both ion modes, which are shown in Figure 1b,c,e,f. A clear separation between the M and C groups and the A and M groups was observed. The parameters for the evaluation of the OPLS-DA models are listed in Table S2. The *R*^2^*X*, *R*^2^*Y*, and *Q*^2^ (cum) values suggested the fitness and prediction of the OPLS-DA model, with no overfitting.

#### 2.2.3. Screening and Identification of Potential Biomarkers from Urine Samples

**Figure 2:**
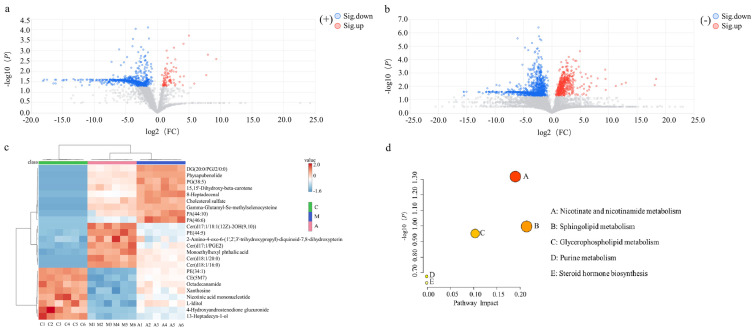
Analysis of differential metabolites of urine samples: volcano plots of the M and A groups in positive (**a**) and negative (**b**) ion modes; heat map of the C, M, and A groups (**c**); KEGG enrichment in metabolic pathways (**d**).

After multivariate statistical analysis of variance, the metabolites with significant differences between groups were screened as biomarkers for AMFP-affected immunosuppressed mice. As shown in Figure 2a,b, compared to the M group, metabolites in the A group exhibited downregulation and upregulation, represented by blue (FC < 1/1.5, *p* < 0.05) and red (FC > 1.5, *p* < 0.05) dots, respectively. Metabolites with VIP ≥ 1.0, *p* < 0.05, and FC > 1.5 or FC < 1/1.5 were selected for structural identification of candidate biomarkers. Twenty-four potential biomarkers were identified using urine metabolomics databases, with their details listed in Table 1, and a comparative analysis of intergroup biomarker changes was performed. Heatmap analysis of the identified differential biomarkers was performed, and the results are presented in Figure 2c. Significant intergroup metabolic differences were observed between the A and M groups, whereas intragroup variations showed high consistency.

#### 2.2.4. Pathway Analysis of Urinary Metabolism

The identified biomarkers contained 12 categories: organooxygen compounds, pyridine nucleotides, glycerophospholipids, carboxylic acids and derivatives, monoethylhexyl phthalate, steroids and derivatives, petrin and derivatives, purine nucleosides, sphingolipids, fatty acyls, isoprenoid lipids, and carboximidic acids and derivatives. These metabolites were annotated to five major metabolic pathways (*p* < 0.05 FDR correction), as shown in Figure 2d, including: nicotinate and nicotinamide metabolism, sphingolipid metabolism, glycerophospholipid metabolism, purine metabolism, and steroid hormone biosynthesis.

### 2.3. Serum Metabolomics Revealed the Immunomodulatory Effects of AMFP

#### 2.3.1. Metabolomics Data Collection from Serum Samples

The BPI chromatograms of the C, M, and A groups of the serum samples analyzed by UPLC-Q-TOF/MS in both positive and negative ion modes are shown in Figure S2. The data were processed as described in Section 3.4.2, and the metabolites were screened.

#### 2.3.2. Multivariate Statistical Analysis of Serum Metabolomics

**Figure 3:**
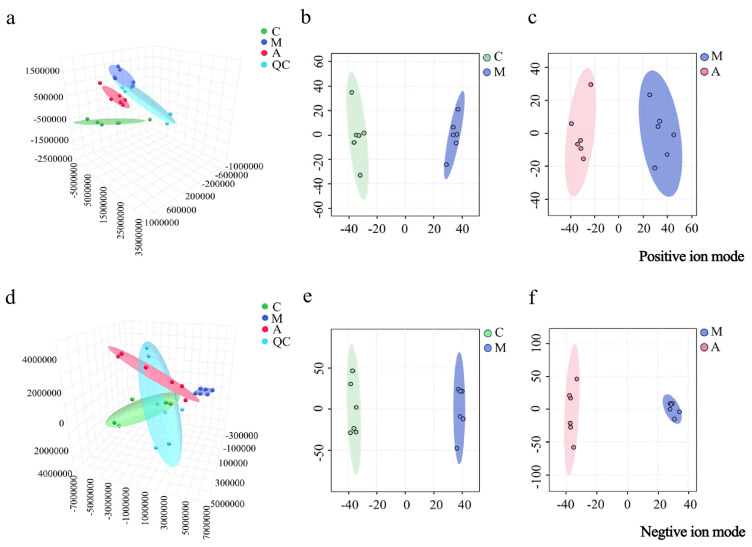
PCA 3D plots of serum samples from the C, M, and A groups in positive (**a**) and negative (**d**) ion modes; OPLS-DA plots of serum samples from the C and M groups in positive (**b**) and negative (**e**) ion modes; OPLS-DA plots of serum samples from the M and A groups in positive (**c**) and negative (**f**) ion modes.

The collected data of the metabolites were imported for PCA and OPLS-DA analysis. In Figure 3a,d, the PCA results demonstrate that the metabolites clustered within the C, M, and A groups, while significant intergroup metabolic differences were identified. In Figure 3b,c,e,f, the OPLS-DA analysis demonstrates a distinct separation between the M and C groups and the A and M groups. From the parameters *R*^2^*X*, *R*^2^*Y*, and *Q*^2^ (cum), values of the OPLS-DA models were evaluated (Table S3), suggesting excellent fitness and prediction.

#### 2.3.3. Screening and Identification of Potential Biomarkers from Serum Samples

**Figure 4:**
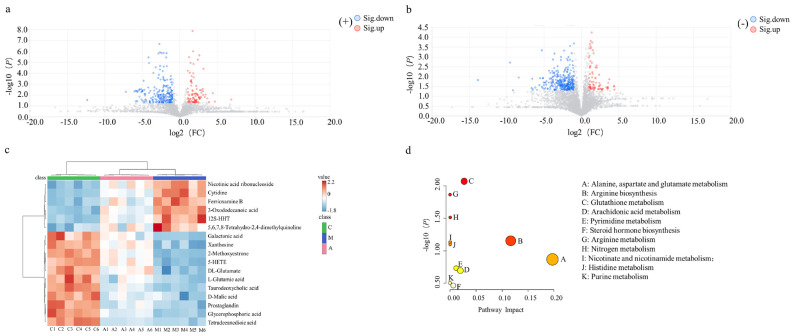
Analysis of differential metabolites of serum samples: volcano plots of the M and A groups in positive (**a**) and negative (**b**) ion modes; heat map of the C, M, and A groups (**c**); KEGG enrichment in metabolic pathways (**d**).

Metabolites with significant differences between groups were screened as biomarkers after multivariate statistical analysis of variance. As shown in the volcano plots (Figure 4a,b), the metabolites that exhibited downregulation (blue (FC < 1/1.5, *p* < 0.05)) and upregulation (red (FC > 1.5, *p* < 0.05)), with VIP ≥ 1.0, were selected for structural identification of candidate biomarkers. The 17 potential biomarkers identified using serum metabolomics databases are listed in Table 2. The identified differential biomarkers that were used to perform a comparative analysis of intergroup changes are presented in the heatmap in Figure 4c.

#### 2.3.4. Pathway Analysis of Serum Metabolism

The identified biomarkers included eight categories: carboxylic acids and derivatives, fatty acyls, quinolines and derivatives, keto acids and derivatives, glycerophospholipids, organooxygen compounds, steroids and derivatives, and pyrimidine nucleosides. These metabolites were annotated to 13 major metabolic pathways (*p* < 0.05 FDR correction) (Figure 4d), including: alanine, aspartate and glutamate metabolism, arginine biosynthesis, glutathione metabolism, arachidonic acid metabolism, pyrimidine metabolism, steroid hormones biosynthesis, arginine metabolism, nitrogen metabolism, nicotinate and nicotinamide metabolism, histidine metabolism, butyrate metabolism, glyoxylate and dicarboxylate metabolism, and purine metabolism.

### 2.4. Lipid Metabolomics Revealed the Immunomodulatory Effects of AMFP

#### 2.4.1. Lipidomics Data Collection

Lipids in serum samples were also analyzed by UPLC-Q-TOF/MS, and the BPI chromatograms in both positive and negative ion modes are shown in Figure S3. There were differences in the lipid metabolites of each group. The data on lipids were further processed and analyzed.

#### 2.4.2. Multivariate Statistical Analysis of Lipid Metabolomics

**Figure 5:**
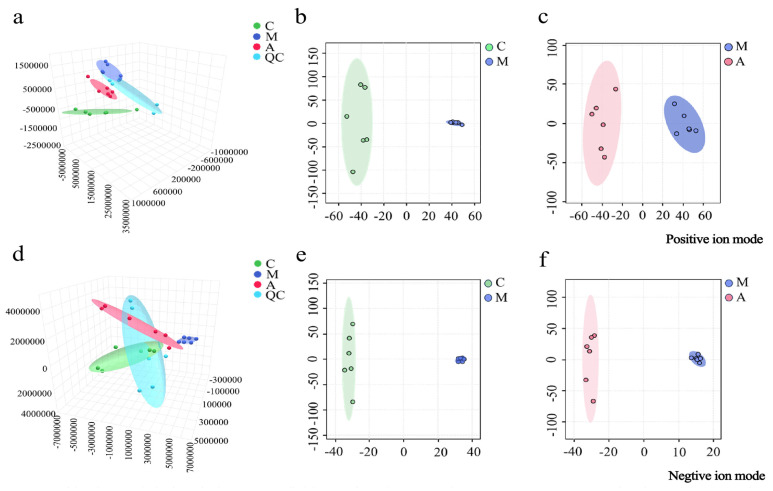
PCA 3D plots of lipids from the C, M, and A groups in positive (**a**) and negative (**d**) ion modes; OPLS-DA plots of lipids from the C and M groups in positive (**b**) and negative (**e**) ion modes; OPLS-DA plots of lipids from the M and A groups in positive (**c**) and negative (**f**) ion modes.

The PCA and OPLS-DA analysis results of the detected lipids are presented in Figure 5. PCA analysis revealed intragroup clustering and distinct intergroup separation. OPLS-DA analysis demonstrated a clear separation between groups, indicating significant alterations in lipid metabolic profiles. No overfitting was found in the validation of the OPLS-DA models (Table S4).

#### 2.4.3. Screening and Identification of Differential Lipid Biomarkers

**Figure 6:**
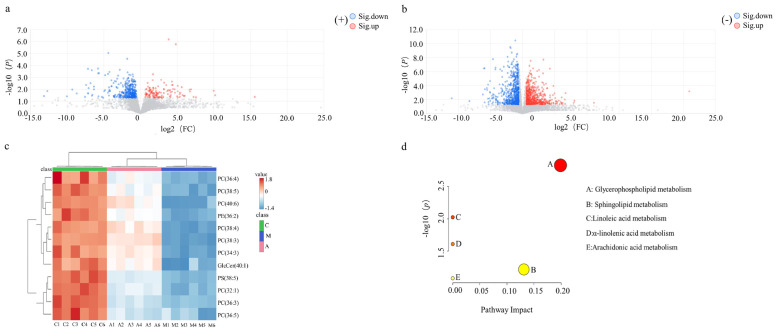
Analysis of differential metabolites of lipids: volcano plots of M and A groups in positive (**a**) and negative (**b**) ion modes; heat map of the C, M, and A groups (**c**); KEGG enrichment in metabolic pathways (**d**).

By multivariate statistical analysis of variance, metabolites with significant differences between groups were screened as biomarkers. The metabolites exhibited downregulation and upregulation, represented by blue (FC < 1/1.5, *p* < 0.05) and red (FC > 1.5, *p* < 0.05) dots, respectively (Figure 6a,b). Metabolites with VIP ≥ 1.0, *p* < 0.05, and FC > 1.5 or FC < 1/1.5 were selected for structural identification of candidate biomarkers. Twelve potential biomarkers were identified using lipid metabolomics databases, and their details are listed in Table 3. A heatmap analysis of the identified differential biomarkers is shown in Figure 6c.

#### 2.4.4. Pathway Analysis of Serum Lipid Metabolism

The 12 identified differential lipid biomarkers were classified into phosphatidylcholines (PCs), sphingolipids (SPLs), phosphatidylserines (PSs), and phosphatidylethanolamines (PEs). These metabolites were annotated to five major metabolic pathways (*p* < 0.05 FDR correction) (Figure 6d), including: glycerophospholipid metabolism, α-linolenic acid metabolism, linoleic acid metabolism, sphingolipid metabolism, and arachidonic acid metabolism.

### 2.5. Combined Metabolomics Analysis of the Immunomodulatory Mechanism of AMFP

#### 2.5.1. Integrated Multi-Omics Metabolic Pathway Analysis

**Figure 7:**
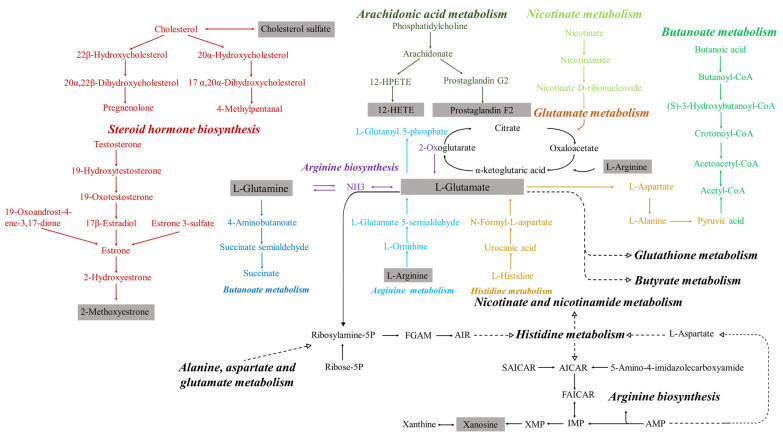
The metabolic pathway scheme of differential endogenous metabolites. The metabolites are identified with a grey-shaded square frame.

Integrated metabolomics and lipidomics analysis revealed the mechanism of AMFP in alleviating metabolic dysregulation in immunosuppressed mice. The urinary, serum, and lipid metabolomics data were combined to screen for common pathways. As shown in Figure 7, differential biomarkers were enriched in 17 metabolic pathways. Among them, nicotinate and nicotinamide metabolism, sphingolipid metabolism, glycerophospholipid metabolism, purine metabolism, steroid hormone biosynthesis, and arachidonic acid metabolism were the common pathways. Those key pathways were mutually validated across multi-omics analyses. And the regulation of their metabolic abnormalities played a key role in improving immune function in the immunosuppressed mice.

#### 2.5.2. Nicotinate and Nicotinamide Metabolism

Nicotinic acid (NA) and nicotinamide (NAM) are two forms of vitamin B3, serving as precursors for the critical cellular cofactor nicotinamide adenine dinucleotide (NAD^+^) in metabolic reactions [22]. During immune responses, immune cells upregulated mitochondrial metabolism and aerobic glycolysis. NAD^+^ played a pivotal role in both metabolic pathways, and elevated intracellular NAD^+^ levels thereby enhanced immune activation [23]. Supplementing NAD^+^ precursors (such as NA and NAM), activating NAD^+^ biosynthetic pathways, modulating NAD^+^-consuming enzymes, and elevating intracellular NAD^+^ levels could enhance the host’s immune response capacity. NAD^+^ acted as an immunomodulator [24] and was capable of promoting the immune homeostasis by suppressing inflammation and preventing inflammation-induced apoptosis [25]. AMCP was likely to enhance immune response capability and improve immune function by elevating endogenous NAD^+^ levels.

#### 2.5.3. Sphingolipid Metabolism

Sphingolipids, as essential structural components of cell membranes, can regulate cellular functions through signal transduction [26,27]. AMFP might participate in regulating T cell activation and proliferation by promoting the generation of sphingolipid metabolite ceramide (Cer), thereby restoring effector T cell function.

#### 2.5.4. Glycerophospholipid Metabolism

Glycerophospholipids, as the major components of cell membranes, can be classified into different categories based on their polar head groups, including PC, PE, etc. PC and LPC serve as crucial membrane components involved in signal transduction and immunomodulation [28]. PC is hydrolyzed to form LPC through catalysis by phospholipase A2. AMFP promoted the activation of quiescent T lymphocytes by enhancing the metabolism of PC to LPC [29]. PE is an abundant membrane phospholipid and serves as an important precursor for PC [30]. Follicular helper T cells (TFH cells) primarily function to assist B cell maturation, germinal center formation, antibody class switching, and somatic hypermutation, serving as a key component in mediating B cell humoral immunity [31]. AMFP regulated the differentiation and development of TFH cells by promoting PE metabolism, thereby influencing humoral immune responses and participating in immunomodulatory processes.

#### 2.5.5. Purine Metabolism

Purine metabolism, involving a series of enzymes and metabolites, plays extensive regulatory roles in immune processes. Hypoxanthine-guanine phosphoribosyltransferase (HGPRT) participated in the regulation of purine metabolism in M2 macrophages [32]. HGPRT promoted tissue repair and immune tolerance while suppressing inflammatory responses [33]. Adenosine deaminase (ADA) reduced adenosine concentrations in local tissues and blood by degrading adenosine, thereby attenuating its immunosuppressive effects and enhancing immune cell activity [34]. The purine metabolite inosine suppressed pro-inflammatory cytokine production and enhanced anti-inflammatory cytokine secretion through activation of adenosine receptors (A1R, A2AR, A2BR, A3R), thereby attenuating inflammatory responses [35]. Inosine mitigated tissue damage and suppressed inflammatory responses by directly reducing neutrophil infiltration and ROS production [36]. AMFP could enhance host immune responsiveness and anti-inflammatory capacity by promoting purine metabolism to elevate levels of HGPRT, ADA, and inosine.

#### 2.5.6. Steroid Hormone Biosynthesis

Steroid hormones, particularly glucocorticoids and androgens, exert significant effects on the immune system. Glucocorticoids enhanced the immune response by inducing the expression of interleukin-7 receptor (IL-7R) and chemokine receptor 4 (CXCR4) in T cells and promoting T cell accumulation in lymphoid organs [37]. Androgens enhanced humoral immunity by suppressing the activation of type 2 innate lymphoid cells (ILC2s), inhibiting the differentiation of T helper 2 (Th2) and T helper 17 (Th17) cells, and augmenting the suppressive function of regulatory T cells (Tregs) [38]. AMFP might enhance host immune responses by promoting the biosynthesis of steroid hormones.

#### 2.5.7. Arachidonic Acid Metabolism

Arachidonic acid (AA) is an ω-6 polyunsaturated fatty acid and serves as a precursor for eicosanoids. AA is oxidized by cyclooxygenase to generate prostaglandin E2 (PGE2). AMCP could promote PGE2 synthesis, which, in turn, suppressed T cell differentiation into T helper 1 (Th1) cells and modulated B cell function [39]. AMCP promoted macrophage polarization toward the alternatively activated phenotype (M2) by enhancing PGE2 synthesis, thereby facilitating their anti-inflammatory functions [40]. This result was consistent with the findings in Section 2.1.4 that AMFP significantly enhanced macrophage phagocytic capacity in the immunocompromised mice. AA is metabolized by lipoxygenase (LOX) to form 5-HETE, which subsequently induces neutrophil aggregation [41]. AMFP also enhanced immune responses by promoting the metabolism of AA to 5-HETE, thereby inducing neutrophil aggregation.

#### 2.5.8. Alanine, Aspartate and Glutamate Metabolism

Alanine generates pyruvate through transamination. Pyruvate is oxidized to acetyl-CoA and enters the TCA cycle, producing ATP for immune cells. Pyruvate suppresses ROS generation, thereby alleviating inflammation and protecting immune cells [42]. Aspartate generates oxaloacetate through transamination. Oxaloacetate enhanced the antioxidant capacity of immune cells through modulation of the NADH/NAD^+^ ratio [43]. In the urea cycle, aspartate reacts with citrulline to form argininosuccinate, which is further converted to arginine. Arginine regulates inflammation and enhances pathogen defense by stimulating nitric oxide (NO) synthesis [44]. Glutamate, cysteine, and glycine combined to synthesize glutathione, which protected against oxidative stress, sustained T-cell functionality, and modulated macrophage polarization through redox signaling regulation [45]. Glutamate is converted to glutamine by glutamine synthetase. Glutamine served as a critical metabolic substrate for T cells, B cells and macrophages and alleviates inflammation through modulation of NF-κB signaling [46]. AMFP could enhance the antioxidant capacity of immune cells by promoting the production of alanine, aspartate, and glutamate metabolites, thereby inhibiting excessive inflammatory responses and maintaining immune homeostasis.

#### 2.5.9. Arginine Biosynthesis

Arginine, as a free amino acid in the body, is a component of most proteins and a substrate for several non-protein nitrogenous compounds, many of which play a role in the immune system [47]. AMFP enhanced the body’s humoral immune function by promoting arginine biosynthesis. The upregulation of arginine biosynthesis stimulated immunoglobulin production and influenced the maturation of pre-B cells [48,49,50]. Meanwhile, T cell proliferation could be increased by elevating specific receptor expression and enhancing IL-2 availability [48,49,50].

#### 2.5.10. Glutathione Metabolism

Glutathione (GSH), the tripeptide of L-glutamate, L-cysteine, and glycine, serves as the key intracellular antioxidant regulating redox homeostasis [51]. GSH’s metabolism in organisms is mainly regulated by enzymes such as glutathione reductase (GSH-GR) and glutathione peroxidase (GSH-Px). GSH produces oxidized glutathione (GSSG) through the catalytic action of GSH-Px. GSSG could be reduced to GSH in the presence of the catalyst GSH-GR and the reducing agent NADPH, thereby protecting the body’s immune system from oxidative damage [52]. Moreover, an increased level of intracellular GSH stimulated interleukin-12 (IL-12) and interleukin-27 (IL-27) production, thereby promoting the differentiation of naive CD4+ T cells into Th1 cells [53]. As shown by the Section 2.1.6 serum GSH-Px activity assay results, AMFP significantly elevated GSH-Px levels, demonstrating potent antioxidant activity. These results further validated that AMFP positively modulated GSH metabolism by enhancing glutathione–peroxide interactions.

#### 2.5.11. Arginine Metabolism

Arginine was catalyzed by arginase to produce urea and ornithine, which was subsequently converted by ornithine decarboxylase into polyamines, thereby downregulating the release of pro-inflammatory cytokines [54]. Simultaneously, arginine could be catalyzed by nitric oxide synthase (NOS) to generate NO and citrulline [55,56]. AMFP might exert immunomodulatory effects by enhancing NO synthesis, which subsequently enhances natural killer (NK) cell activity, activates peripheral blood monocytes, and regulates cytokine secretion by T lymphocytes and macrophages [48].

#### 2.5.12. Nitrogen Metabolism

Nitrogen metabolism represents one of the essential biochemical processes in the human body, primarily involving protein synthesis, degradation, and transport. The key processes of nitrogen metabolic pathways include amino acid metabolism, the urea cycle, and the glutamine cycle, all of which play crucial roles in maintaining systemic nitrogen balance.

Amino acid metabolism supplied essential nitrogen sources for immune cells and supported their proliferation and activation [57]. In immune regulation, efficient urea cycle metabolism was critical for maintaining the development and long-term survival of memory T cells, playing a pivotal role in stabilizing the intracellular and extracellular microenvironment of immune cells [58]. Glutamine served as a crucial energy source for lymphocytes and macrophages. The glutamine cycle indirectly modulated immune cell activity and function by regulating glutamine availability, which was essential for maintaining normal immune system function [59]. AMFP enhanced human immunity and reduced infection/disease risks by promoting nitrogen metabolism and maintaining optimal nitrogen balance.

#### 2.5.13. α-Linolenic Acid Metabolism

α-Linolenic acid (ALA) is an essential fatty acid indispensable for humans. Studies demonstrated that docosahexaenoic acid (DHA), a metabolic derivative of ALA, exerted positive immunomodulatory effects. DHA enhanced macrophage proliferation and phagocytic activity, stimulated splenocyte proliferation, elevated NK cell cytotoxicity, and promoted cytokine production in vivo [60]. AMFP enhanced immune function by promoting ALA metabolism and elevating DHA levels in vivo. These findings were consistent with those in Section 2.1.1 and Section 2.1.4, which showed that AMFP significantly enhanced the immune organ index and macrophage phagocytic capacity in immunocompromised mice.

#### 2.5.14. Linoleic Acid Metabolism

Glyoxylate and dicarboxylates serve as crucial metabolic intermediates, participating in diverse biochemical processes including energy metabolism, detoxification, and anabolic pathways. Glyoxylate is primarily generated in the glyoxylate cycle through the cleavage of isocitrate by isocitrate-lyase, with its key metabolites including glycine and malate. Dicarboxylates participate in the TCA cycle, fatty acid β-oxidation, and amino acid metabolism. Key dicarboxylate metabolites include oxaloacetate, malate, and related intermediates. AMCP could enhance the anti-inflammatory and reparative functions of M2 macrophages by promoting malate production, thereby maintaining an intact TCA cycle [61]. AMFP also promoted oxaloacetate production, replenished TCA cycle intermediates, and supported the metabolic requirements of M2 macrophages and regulatory T cells (Tregs) [62]. AMFP was likely to suppress the activity of M1 macrophages and pro-inflammatory Th17 cells while reducing the release of inflammatory cytokines (IL-6, IL-1β) by promoting oxaloacetate generation [63].

This study presented a multi-omics approach by integrating metabolomics and lipidomics data to elucidate the immunomodulatory mechanisms of AMFP. Multivariate statistical tools are among the most widely employed techniques in metabolomics data analysis for extracting latent variables in either an unsupervised or supervised manner. PCA is a highly practical unsupervised method that reduces the complexity or dimensionality of a dataset to a more manageable two or three dimensions by extracting the primary sources of variation, without prior knowledge of sample classifications. PCA is particularly useful for detecting outliers and identifying inherent clusters within sample groups, thereby aiding in the recognition of similar biological characteristics. On the other hand, OPLS-DA is a commonly used supervised approach, frequently applied to discern biomarkers and distinctions among different sample groups. In this study, the OPLS-DA models of urinary, serum, and lipid metabolomics were validated by *R*^2^*X*, *R*^2^*Y*, and *Q*^2^ (cum) values. The results suggested excellent fitness and prediction with no overfitting. The integrated analysis revealed that there were overlaps in the metabolic pathways involving the biomarkers identified in the urinary, serum, and lipid metabolomics. The results demonstrated that the multi-omics data were interconnected and cross-validated, which jointly elucidated AMFP’s significant impact on the metabolic pathways regulating immune system functions.

## 3. Materials and Methods

### 3.1. Reagents and Materials

Physiological saline was purchased from Shandong Hualu Pharmaceutical Co., Ltd. (Liaocheng, China). Phosphate buffer saline (HPO_4_^2−^/H_2_PO_4_^−^, pH 7.2) and 2,6-2,6-di-tert-butyl-4-methylphenol were purchased from Shanghai Yuanye Bio-Technology Co., Ltd. (Shanghai, China). Cyclophosphamide for injection was purchased from Jiangsu Hengrui Pharmaceuticals Co., Ltd. (Lianyungang, China). RPMI-1640 Medium and fetal bovine serum were purchased from HyClone Laboratories (Shanghai, China). Concanavalin A was purchased from Sigma (Shanghai, China). Hydrochloric acid (3 mol·L^−1^, pH 7.0) was purchased from Tianjin Xintong Fine Chemical Co., Ltd. (Tianjin, China). Penicillin–Streptomycin Solution was purchased from Beyotime Biotech Inc. (Shanghai, China). IL-2 and IFN-γ ELISA Kits, malondialdehyde (MDA), superoxide dismutase (SOD), and glutathione peroxidase (GSH-Px) kits were purchased from Nanjing Jiancheng Bioengineering Institute (Nanjing, China). Methyl tert-butyl ether was purchased from Beijing Chemical Works (Beijing, China). Sodium carbonate, acetone, sesame oil, 2,4-dinitrofluorobenzene (DNFB), and 95% ethanol (*v*/*v*) were purchased from Sinopharm Chemical Reagent Co., Ltd. (Shanghai, China). Acetonitrile, isopropanol, and methanol (HPLC grade) were purchased from Thermo Fisher Scientific Inc. (Changchun, China).

The AMFP was prepared following the published process of our group [17]. This polysaccharide was extracted with water by ultrasonics, then decolorized and deproteinated. The purification was performed on a cellulose column. It was indicated to have a high degree of purity, without impurities such as proteins, nucleic acids, and polypeptides. D-arabinose and D-xylose were the main components of AMFP, which contained both β-type and α-type glycosidic bonds with a molecular weight of 83,444 Da.

### 3.2. Instruments

The instruments used in this study included the following: UPLC-Q-TOF/MS (Synapt G2-Si, Waters Corporation, Milford, CT, USA); an ACOUITY UPLC BEHC8 chromatography column (1.7 μm, 2.1 mm × 50 mm, Waters Corporation, Beijing, China); an MEX-7222K fully automated hemocyte analyzer (Nihon Kohden Corporation, Tokyo, Japan); a Milli-Q ultrapure water preparation instrument (Merck Millipore, Shanghai, China); an XS-204 electronic analytical balance (METTLER TOLEDO, Shenyang, China); an XM-P22H ultrasonic cleaning machine (Xiaomei Ultrasound Instrument Co., Ltd., Kunshan, China); an ultra-low temperature freezer (Zhongke Meiling Cryogenics Co., Ltd., Hefei, China); a TDL-5000B Centrifuge (Shanghai Anting Scientific Instrument Factory, Shanghai, China); a microplate reader (Bio-Rad Laboratories, Inc., Shanghai, China); and a carbon dioxide incubator (Thermo Fisher Scientific Inc., Beijing, China).

### 3.3. Animal Experiment

ICR mice (male, 4–5 weeks, 20 ± 2 g) were adaptively fed for 3 days in the SPF environment of the Animal Experiment Center of Changchun University of Chinese Medicine. The mice were randomly divided into control (C), model (M), and AMFP (A) groups (*n* = 15). The M and A groups were induced by intraperitoneal injection of cyclophosphamide (80 mg·kg^−1^·d^−1^) for three days. Then, the cyclophosphamide injection was repeated every 5 days for four times for immunosuppression modeling. Meanwhile, the C group was given the same volume of saline. From the fourth day, the A group was gavaged AMFP (400 mg·kg^−1^·d^−1^) for the following 21 days. For the AMFP dose optimization and selection performed, please refer to a previous publication by our group [17]. The C and M groups were gavaged with the same volume of saline consecutively. After the final administration, the mice were fasted for 12 h with free access to water. And urine, blood and organ samples were collected and stored in the −80 °C freezer. The animal protocols were approved by the Animal Ethics Committee of Changchun University of Chinese Medicine (2023228). The evaluation method for the immunomodulatory effect of polysaccharides was implemented in accordance with “Functional Inspection and Evaluation Methods for Health Food (2023 Edition)” [64], issued by the State Administration for Market Regulation of China, and followed the methods and evaluation standards for enhancing immunity as stipulated therein.

#### 3.3.1. Immune Organ Index Measurement

The spleens and thymuses of the mice were immediately excised, rinsed with saline, blotted dry with filter paper, and precisely weighed. The immune organ indices were calculated using the following formulae:

Spleens index (mg·g^−1^) = spleens weight (mg)/body weight (g) (1)

Thymuses index (mg·g^−1^) = thymuses weight (mg)/body weight (g) (2)

#### 3.3.2. Delayed-Type Hypersensitivity

First, 50 mg of DNFB was accurately weighed into a clean, dry vial. Then, 5 mL of freshly prepared acetone–corn oil solution (acetone–corn oil = 1:1, *v*/*v*) was added to the vial. The vial was capped and sealed with tape. The mixture was vortexed thoroughly, and samples were drawn using a 250 μL syringe through the cap.

A total of 10 μL of DNFB solution was evenly applied to both sides of the right ear of the mice. After 24 h, the mice were euthanized by cervical dislocation, and both the left and right ear pinnae were excised. An 8 mm diameter ear piece was removed with a hole puncher, and the degree of delayed-type hypersensitivity was indicated by the degree of auricle swelling.

Auricle swelling degree (mg) = right ear weight (mg) − left ear weight (mg)(3)

#### 3.3.3. Determination of Peripheral Blood Parameters

After the blood was collected intraocularly, an appropriate amount of plasma was placed in EDTA anticoagulant tubes. The white blood cell, red blood cell, and platelet counts and the percentages of neutrophils, lymphocytes, and monocytes were determined using an automatic hemocyte analyzer.

#### 3.3.4. Carbon Clearance Assay

The mice were intravenously injected with India ink (10 mL·kg^−1^, 4-fold saline-diluted) via the tail, and the timing was initiated immediately. At 2 min (t_1_) and 10 min (t_2_) post-injection, 20 μL of blood was collected from the retro-orbital venous plexus of each mouse and immediately mixed with 2 mL sodium carbonate solution (1%, *w*/*v*). The optical density (OD) was measured at 600 nm using a microplate reader. The mice were euthanized by cervical dislocation. Their livers and spleens were excised, blotted dry on filter paper to remove surface blood, and weighed separately. The phagocytic capacity of macrophages was represented by the phagocytic index, calculated using the following formula:

Carbon clearance index (K) = (lgOD_1_ − lgOD_2_)/(t_2_ − t_1_) (4)

Phagocytic index = ∛K × body weight/(liver weight + spleen weight)(5)

#### 3.3.5. Determination of the Immune Cytokines IL-2 and IFN-γ

An appropriate amount of plasma was centrifuged at 2000 rpm for 10 min. The supernatant serum was collected, and the contents of IL-2 and IFN-γ were measured according to the kit’s instructions.

#### 3.3.6. Determination of Antioxidant Activity

An appropriate amount of plasma was centrifuged at 2000 rpm for 10 min. The supernatant serum was collected. The contents of malondialdehyde (MDA) and the activities of superoxide dismutase (SOD) and glutathione peroxidase (GSH-Px) were measured according to the kit’s instructions.

#### 3.3.7. Detection of Splenic Lymphocyte Proliferation

A spleen sample was filtered through a 200-mesh steel sieve to prepare a single-cell suspension in PBS and centrifuged at 1000 rpm for 5 min; the supernatant was discarded. Then, 2 mL of red blood cell lysis buffer was added, and the cells were resuspended, incubated for 5 min, and centrifuged at 1000 rpm for 5 min. The supernatant was discarded. The cells were resuspended in complete RPMI-1640 medium, adjusted to an appropriate concentration, and cultured at 37 °C with 5% CO_2_ for 2 h. The cell suspension was collected from the culture flasks, discarding the adherent monocytes on the flask walls. The remaining cells were splenic lymphocytes, which were adjusted to a concentration of 2 × 10^6^ cells·mL^−1^.

The proliferative activity of the splenic lymphocytes was measured using a CCK-8 kit. The cell suspension was added to 96-well plates at 100 μL per well. Then, 20 μg·mL^−1^ LPS was added to the LPS-stimulated wells, and 10 μg·mL^−1^ ConA was added to the ConA-stimulated wells. The cells were cultured at 37 °C with 5% CO_2_ for 72 h. Then, 10 μL/well of CCK-8 solution was added, followed by a 30 min incubation. Absorbance was measured at 450 nm using a microplate reader. The lymphocyte proliferation level was evaluated using the cell activity index as the indicator.

Cell activity index (%) = [(D^1^_450nm_ − D^2^_450nm_)/D^2^_450nm_] × 100% (6)

D^1^_450nm_, absorbance of stimulated cells; D^2^_450nm_, absorbance of unstimulated cells.

#### 3.3.8. Statistical Methods

Statistical analysis was performed using SPSS 23.0 software. Experimental results are presented as mean ± standard deviation (SD). Differences between groups were analyzed by ANOVA with considered statistically significant.

### 3.4. Metabolomics Analysis

#### 3.4.1. Urine Metabolomics Analysis

Urine samples were retrieved from the ultra-low temperature freezer, thawed at 4 °C, and centrifuged at 7000 rpm for 10 min. The supernatant was filtered through a 0.22 μm membrane for UPLC-Q-TOF/MS analysis.

The chromatographic mobile phases were acetonitrile (A) and 0.1% formic acid/H_2_O (*v*/*v*) (B). The gradient elution program was: 0–3 min, 5–20% A; 3–6 min, 20–40% A; 6–8 min, 40–50% A; 8–9 min, 50–80% A; 9–12 min, 80–100% A; and 12–15 min, 100% A. The flow rate was 0.3 mL·min^−1^. The injection volume was 5 μL. The sample plate temperature was 4 °C. The column temperature was 35 °C.

The mass spectrometer was calibrated using sodium formate solution. For real-time calibration, leucine enkephalin was used for mass spectrometry acquisition at a flow rate of 10 μL·min^−1^ with an acquisition frequency of 20 s/time. MS^E^ data were acquired in full scan mode with a mass range of 50–2000 u and a scan time of 0.2 s.

The ESI source was used in both positive and negative ion modes at a temperature of 120 °C. The cone voltage was 40 V, and the extracted cone voltage was 5.0 V. The desolvation gas temperature was 400 °C, with a flow rate of 800 L·h^−1^. The cone gas flow rate was 30 L·h^−1^. The low and high collision energies were 5 eV and 20–40 eV, respectively.

#### 3.4.2. Serum Metabolomics Analysis

Serum samples were retrieved from the ultra-low temperature freezer, thawed at 4 °C, and centrifuged at 10,000 rpm for 10 min. The supernatant was filtered through a 0.22 μm membrane for UPLC-Q-TOF/MS analysis.

The chromatography and mass spectrometry conditions were the same as those in Section 3.4.1.

#### 3.4.3. Serum Lipidomics Analysis

First, 1.5 mL of methanol/MTBE/water (1:5:2, *v*/*v*/*v*) was added to 200 μL of serum. After vortex mixing for 5 min, the mixture was incubated at room temperature for 10 min, then stored at −20 °C overnight for protein precipitation. Then, the samples were centrifuged at 10,000 rpm for 20 min. The organic phase was collected, dried under nitrogen gas, and redissolved in 1 mL of isopropanol/acetonitrile (1:1, *v*/*v*). After filtration through a 0.22 μm membrane, the extracts were analyzed by UPLC-Q-TOF/MS.

Chromatographic mobile phases were acetonitrile/isopropanol (1:1, *v*/*v*) (A) and 0.1% formic acid/H_2_O (*v*/*v*) (B), both containing 10 mmol·L^−1^ ammonium formate. The gradient elution program was: 0–6 min, 5–82% A; 6–7 min, 82–85% A; 7–20 min, 85–90% A; 20–22 min, 90–100% A; and 22–28 min, 100% A. The flow rate was 0.3 mL·min^−1^. The injection volume was 5 μL. The sample tray temperature was 4 °C. The column temperature was 35 °C.

The mass spectrometry conditions were the same as those in Section 3.4.1.

#### 3.4.4. Metabolomics Data Analysis

For quality assurance, equal volumes of all individual specimens were combined to generate pooled QC samples. The instrument analysis started with 10 QC samples. Subsequently, the QC sample was analyzed every 10 test samples to assess system stability, precision, and reproducibility.

The collected metabolomics data were analyzed using Progenesis QI software (3.0.7927.47290), including data import, peak picking, and deconvolution. The data were imported into SIMCA-P 14.0 for multivariate statistical analysis, and principal component analysis (PCA) and orthogonal partial least squares discriminant analysis (OPLS-DA) were performed to preliminarily identify intergroup differences. Potential biomarkers were screened using the three criteria: VIP value (VIP > 1), *t*-test (*p* < 0.05), and fold change (Fc) > 1.50 or < 1/1.50.

The identification of biomarkers based on their accurate masses and MS/MS product ion analysis was compared with the corresponding database resources. The biomarkers in urine and serum were used for the HMDB biochemical database. The LIPID MAPS biochemical database was used for lipid biomarker searches. Metabolic pathways were analyzed using KEGG and Metaboanalyst 4.0.

## 4. Conclusions

This study employed integrated urine metabolomics, serum metabolomics, and lipidomics to analyze the alleviating effects of AMFP on cyclophosphamide-induced immunocompromised mice. Integrated multi-omics analysis revealed that AMFP primarily enhanced both humoral and cellular immune responses in mice by activating immune cells (lymphocytes, macrophages, and natural killer cells) through regulation of immune-metabolic pathways, including nicotinate and nicotinamide metabolism, sphingolipid metabolism, glycerophospholipid metabolism, purine metabolism, steroid hormone biosynthesis, and arachidonic acid metabolism. Furthermore, AMFP enhanced antioxidant enzyme activity and modulated the glutathione metabolic pathway, thereby protecting the immune system from oxidative damage and consequently improving immune function.

This study comprehensively elucidated the immunomodulatory mechanisms of crude polysaccharides from *Aronia melanocarpa* fruit from the perspectives of metabolomics and lipidomics, and further validated the findings of our previous research by metagenomics. These studies fully explored the potential of *Aronia melanocarpa* fruit in enhancing the body’s immunity as well as in preventing and treating diseases. Thereby, this provides theoretical support and technical guidance for the development of *Aronia melanocarpa* fruit as a natural functional resource.

## Figures and Tables

**Table 1:** Differential biomarkers screened out and identified in urine metabolomics.

Rt/min	*m*/*z*	Compounds	Molecular Formula	M/C	A/M
8.88	181.0710	L-iditol	C_6_H_14_O_6_	Down *	Up *
8.88	357.0229	Nicotinic acid mononucleotide	C_11_H_15_NO_9_P	Down *	Up *
8.90	850.6327	PE(44:5)	C_49_H_88_NO_8_P	Up *	Down *
9.39	293.0025	Glutamyl-Se-methylselenocysteine	C_9_H_16_N_2_O_5_Se	Down	Up *
9.55	618.4773	Cer(d17:1/PGE2)	C_37_H_65_NO_6_	Down **	Up **
9.84	279.1560	Monoethylhexyl phthalic acid	C_16_H_22_O_4_	Up *	Down *
9.87	499.1997	4-Hydroxyandrostenedione glucuronide	C_25_H_34_O_9_	Down *	Up **
9.90	817.5033	PG(38:5)	C_44_H_77_O_10_P	Down	Up *
9.93	288.0929	2-Amino-4-oxo-6-(1’,2’,3’-trihydroxypropyl)-diquinoid-7,8-dihydroxypterin	C_9_H_15_N_5_O_6_	Down	Up *
10.23	549.2487	Physapubenolide	C_30_H_40_O_8_	Down	Up *
10.24	265.0568	Xanthosine	C_10_H_12_N_4_O_6_	Down *	Up *
10.46	574.5623	Cer(d38:1)	C_38_H_75_NO_3_	Down	Up *
10.73	297.2423	8-Heptadecenal	C_17_H_32_O	Down	Up *
10.73	252.2441	13-Heptadecyn-1-ol	C_17_H_32_O	Down **	Up *
11.48	623.5361	Cer(d35:1(12Z)-2OH(9,10))	C_35_H_67_NO_5_	Up	Down *
11.85	649.5508	CE(5M7)	C_44_H_72_O_3_	Down *	Up **
12.26	634.4531	15,15’-Dihydroxy-carotene	C_40_H_58_O_2_	Down	Up *
12.37	718.5393	PE(34:1)	C_39_H_76_NO_8_P	Down *	Up *
12.37	744.5702	DG(20:0/PGJ2/0:0)	C_43_H_74_O_7_	Down	Up **
12.44	538.5221	Cer(d34:1)	C_34_H_67_NO_3_	Down	Up *
12.80	284.2950	Octadecanamide	C_18_H_37_NO	Down **	Up *
13.22	465.3032	Cholesterol sulfate	C_27_H_46_O_4_S	Down	Up *
13.22	853.5720	PA(46:6)	C_49_H_85_O_8_P	Down	Up *
13.23	841.5114	PA(44:10)	C_47_H_73_O_8_P	Down	Up *

* *p* < 0.05; ** *p* < 0.01.

**Table 2:** Differential biomarkers screened out and identified in serum metabolomics.

Rt/min	*m*/*z*	Compounds	Molecular Formula	M/C	A/M
0.90	128.0351	L-Glutamic acid	C_5_H_9_NO_4_	Down *	Up *
1.40	177.0390	Galactonic acid	C_6_H_12_O_7_	Down **	Up **
1.41	133.0209	D-Malic acid	C_4_H_6_O_5_	Down **	Up *
4.17	165.8870	DL-Glutamate	C_5_H_9_NO_4_	Down *	Up *
5.78	283.0682	Xanthosine	C_10_H_12_N_4_O_6_	Down **	Up *
5.87	372.2741	Prostaglandin	C_20_H_34_O_5_	Down *	Up *
8.02	237.0615	Nicotinic acid ribonucleoside	C_11_H_14_NO_6_	Up *	Down *
9.09	498.2889	Taurodeoxycholic acid	C_26_H_45_NO_6_S	Down **	Up *
10.41	195.1384	3-Oxododecanoic acid	C_12_H_22_O_3_	Up **	Down **
10.43	172.0737	Glycerophosphoric acid	C_3_H_9_O_6_P	Down *	Up *
10.55	239.1646	Tetradecanedioic acid	C_14_H_26_O_4_	Down *	Up *
10.56	279.1969	12S-HHT	C_17_H_28_O_3_	Up **	Down *
10.62	184.1081	5,6,7,8-Tetrahydro-2,4-dimethylquinoline	C_11_H_15_N	Up **	Down **
11.03	242.0870	Cytidine	C_9_H_13_N_3_O_5_	Up **	Down **
11.08	612.2609	Ferrioxamine B	C_25_H_45_FeN_6_O_8_	Up **	Down **
11.13	321.1473	2-Methoxyestrone	C_19_H_24_O_3_	Down **	Up **
11.30	319.2345	5-HETE	C_20_H_32_O_3_	Down **	Up *

* *p* < 0.05; ** *p* < 0.01.

**Table 3:** Differential lipid biomarkers screened out and identified in lipid metabolomics.

Rt/min	*m*/*z*	Compounds	Molecular Formula	M/C	A/M
8.74	772.5472	PC(36:3)	C_44_H_86_NO_7_P	Down **	Up *
8.91	784.5431	GlcCer(40:1)	C_46_H_89_NO_8_	Down **	Up *
17.92	780.5148	PC(36:5)	C_44_H_78_NO_8_P	Down **	Up *
18.48	782.5305	PC(36:4)	C_44_H_80_NO_8_P	Down **	Up *
19.53	792.5478	PS(38:5)	C_46_H_82_NO_7_P	Down **	Up *
19.59	756.5158	PC(34:3)	C_42_H_78_NO_8_P	Down **	Up *
19.73	744.5204	PE(36:2)	C_42_H_82_NO_7_P	Down **	Up **
21.22	796.5690	PC(38:3)	C_46_H_86_NO_7_P	Down **	Up *
21.39	732.5191	PC(32:1)	C_40_H_78_NO_8_P	Down **	Up *
21.52	808.5429	PC(38:5)	C_46_H_82_NO_8_P	Down **	Up *
21.61	834.5463	PC(40:6)	C_48_H_84_NO_8_P	Down **	Up *
28.78	810.5552	PC(38:4)	C_46_H_84_NO_8_P	Down **	Up *

* *p* < 0.05; ** *p* < 0.01.

## Data Availability

Data are contained within this article and the Supplementary Materials.

## Supplementary Materials

The following supporting information can be downloaded at https://www.mdpi.com/article/10.3390/molecules31071166/s1. Table S1: Modulatory effects of AMCP on the immune regulatory indices in immunosuppressed mice; Table S2: Parameters of the OPLS-DA model of urinary metabolomics; Table S3: Parameters of the OPLS-DA model of serum metabolomics. Table S4: Parameters of the OPLS-DA model of lipid metabolomics. Figure S1: BPI chromatogram of urine samples of the C, M, and A groups; Figure S2: BPI chromatogram of serum samples of the C, M, and A groups; Figure S3: BPI chromatogram of serum lipid samples of the C, M, and A groups.
