# Supplementary material for: Immune Regulatory and Underlying Mechanisms of Polysaccharides from Aronia melanocarpa Fruit by Integrated Analysis of Multiple Endogenous Metabolism
Source: Molecules. 2026 Apr 1;31(7):1166. doi: 10.3390/molecules31071166 (PMC13074254; doi:10.3390/molecules31071166)
Supplement: Supplementary file 1 [file molecules-31-01166-s001.zip › molecules-4150601-supplementary.pdf]

Table S1 Modulatory effects of AMCP on the immune regulatory indices in immunosuppressed mice. ($\bar{x}\pm s$, n=6)

Indices		C group	M group	A group
Body weight		25.57±1.70	21.22±0.88#	23.36±1.03*
Spleen index		4.09±0.34	3.22±0.36#	3.69±0.41*
Thymus index		2.03±0.12	1.16±0.15#	1.86±0.09*
Auricle swelling degree		8.48±1.21	14.26±1.40#	9.34±1.42**
WBC/$\times10^{9}\cdot L^{-1}$		9.58±0.23	6.74±0.09#	8.86±0.06*
PLT/$\times10^{9}\cdot L^{-1}$		765±0.13	567±0.80#	783±0.10*
RBC/$\times10^{12}\cdot L^{-1}$		12.14±0.10	7.27±0.14#	8.33±0.11*
Gran%		15.98±0.12	10.90±0.07#	13.66±0.12*
Lymph%		78.28±0.12	67.73±0.17#	75.98±0.09*
Mon%		4.36±0.12	2.30±0.07#	3.66±0.10*
Carbon clearance index		0.04±0.01	0.01±0.01#	0.03±0.01*
Phagocytic index		7.98±1.62	3.37±1.24#	6.92±1.27*
IL-2 concentration/$pg\cdot mL^{-1}$		112.86±6.32	102.66±5.23##	116.34±5.75*
IFN-γ concentration/$ng\cdot mL^{-1}$		110.33±5.28	86.32±4.43#	103.56±5.76**
MDA/$nmol\cdot mg^{-1}$		129.28±12.33	173.89±16.51#	138.38±13.65**
SOD/$U\cdot mg^{-1}$		133.36±15.78	95.93±12.34##	123.21±13.45**
GSH-Px/$U\cdot mg^{-1}$		89.78±7.82	74.54±7.22##	87.16±6.34**
Cell activity indexes of splenic lymphocytes	No-inducer group	0.32±0.02	0.27±0.02##	0.40±0.01*
	ConA-induced group	0.51±0.01	0.29±0.03##	0.58±0.02*

Note: Compared with C group, #p<0.05, ##p<0.01; Compared with M group, *p<0.05, **p<0.01.

Table S2 Parameters of the OPLS-DA model of urinary metabolomics.

	M *vs* C groups			A *vs* M groups		
**Ion mode**	R^2X	R^2Y	Q^2 (cum)	R^2X	R^2Y	Q^2 (cum)
Positive	0.798	0.946	0.788	0.885	0.980	0.711
Negative	0.739	0.984	0.910	0.850	1.000	0.906

Table S3 Parameters of the OPLS-DA model of serum metabolomics.

	M *vs* C groups			A *vs* M groups		
**Ion mode**	R^2X	R^2Y	Q^2 (cum)	R^2X	R^2Y	Q^2 (cum)
Positive	0.756	0.989	0.928	0.946	0.998	0.941
Negative	0.509	0.9112	0.762	0.724	0.939	0.834

Table S4 Parameters of the OPLS-DA model of lipids metabolomics.

	M *vs* C groups			A *vs* M groups		
**Ion mode**	R^2X	R^2Y	Q^2 (cum)	R^2X	R^2Y	Q^2 (cum)
Positive	0.766	0.995	0.872	0.796	0.979	0.893
Negative	0.941	0.998	0.921	0.715	0.989	0.947

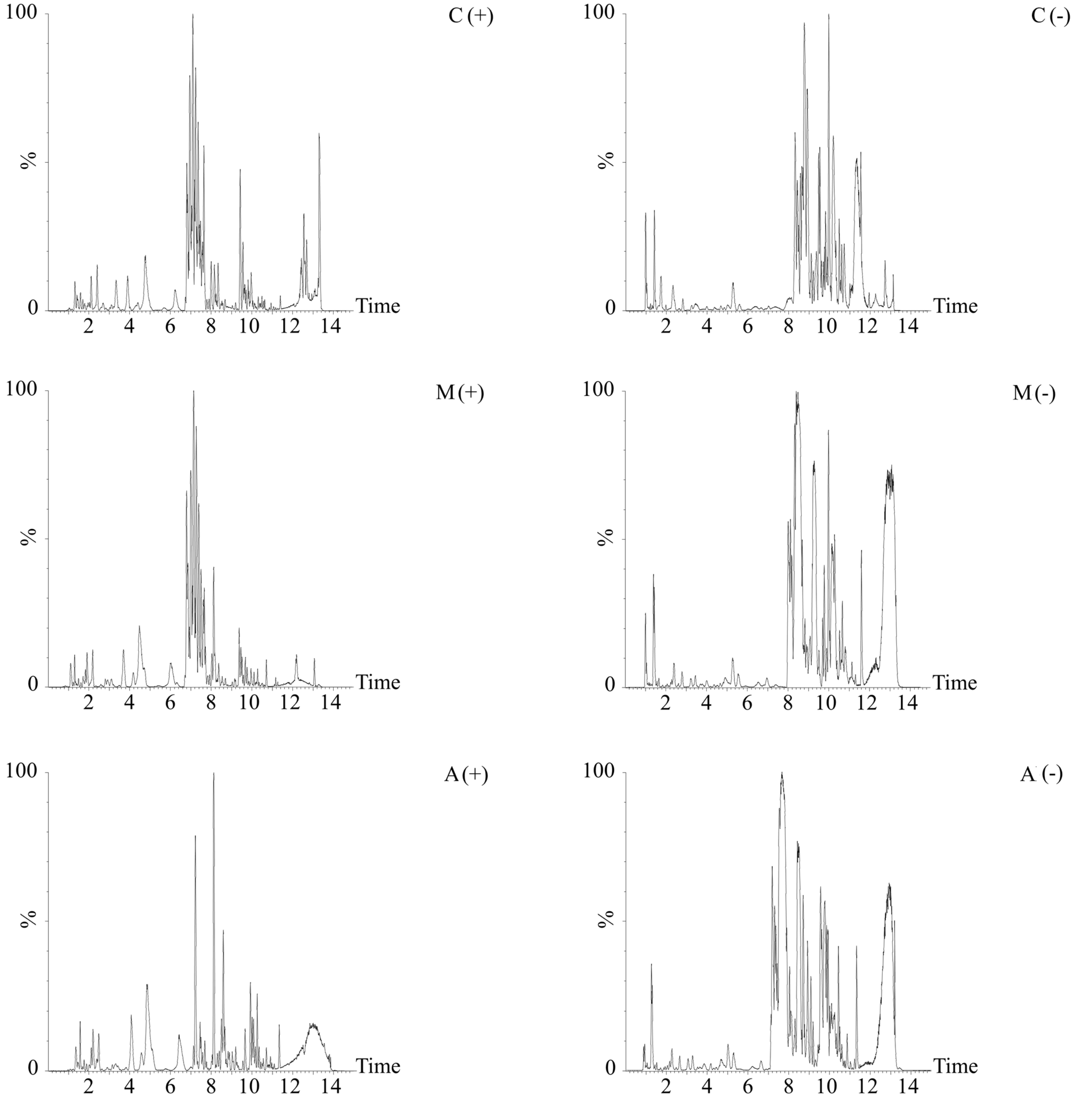

Figure S1 BPI chromatogram of urine samples of C, M, and A groups.

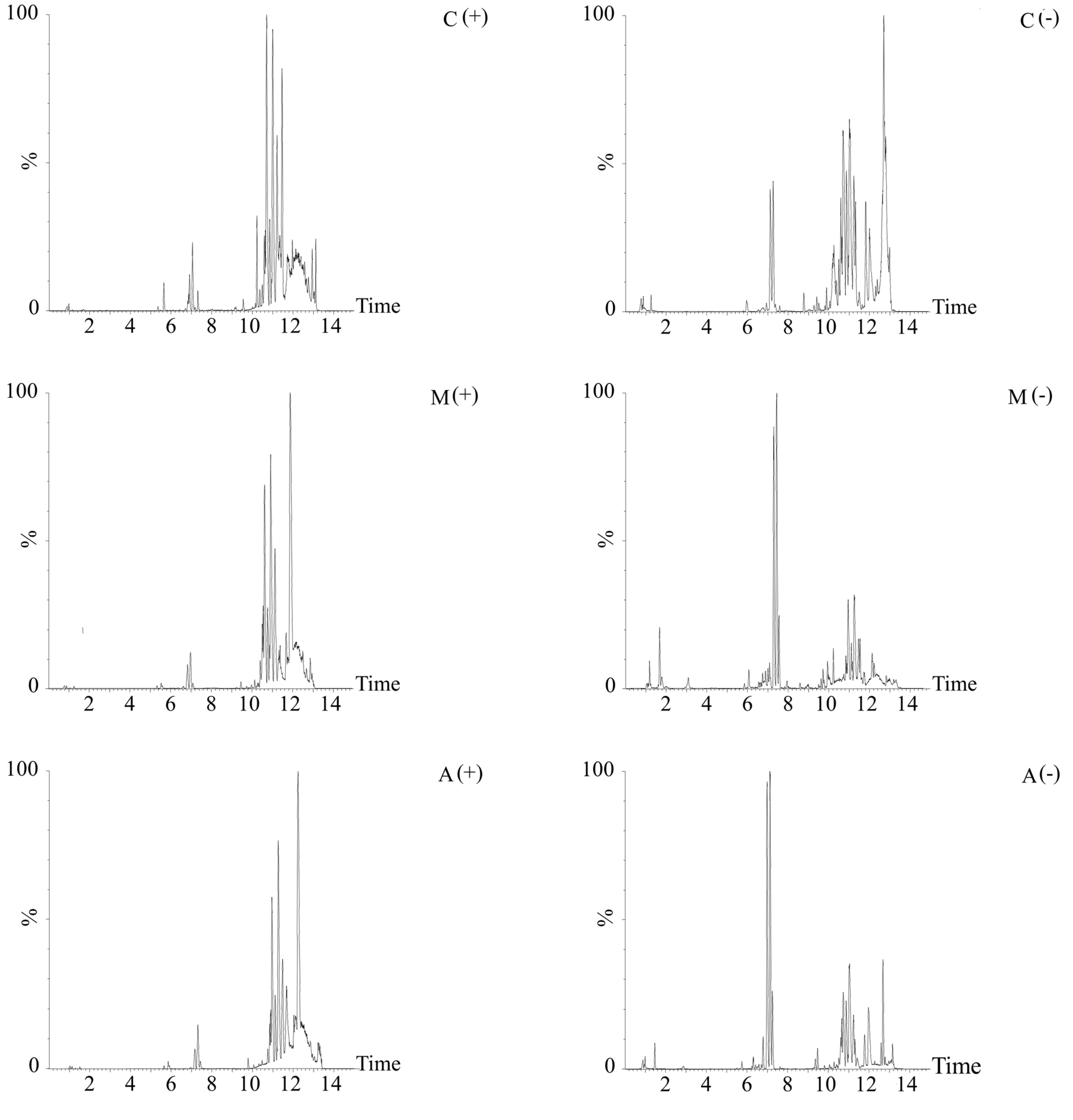

Figure S2 BPI chromatogram of serum samples of C, M, and A groups.

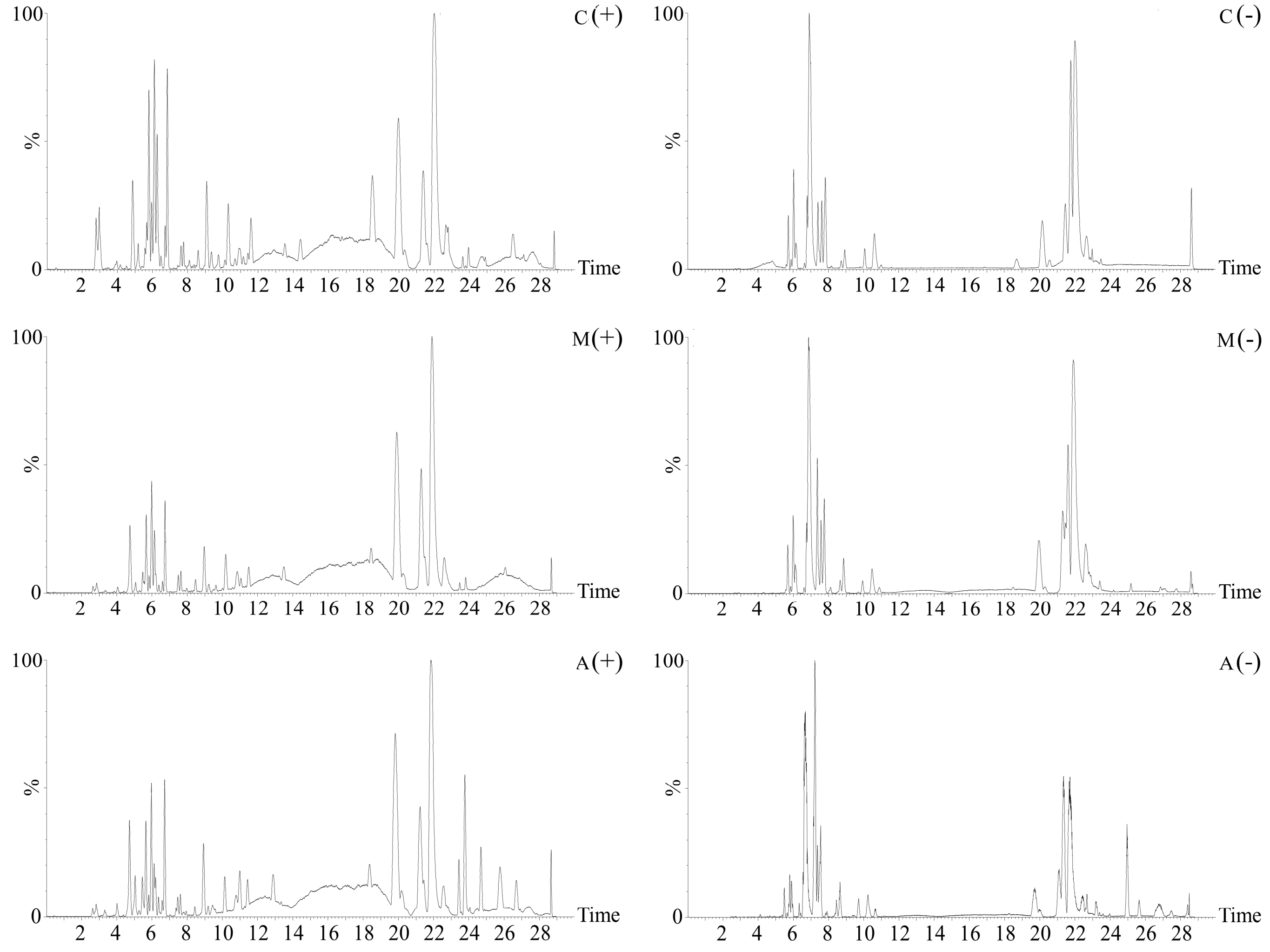

Figure S3 BPI chromatogram of serum lipids samples of C, M, and A groups.
